# Bridges of perspectives: representation of people with lived experience of spinal cord injury in editorial boards and peer review

**DOI:** 10.1186/s41073-023-00138-0

**Published:** 2023-09-21

**Authors:** Anna Nuechterlein, Tanya Barretto, Alaa Yehia, Judy Illes

**Affiliations:** Neuroethics Canada, Division of Neurology, Department of Medicine, University of British Columbia, 2211 Wesbrook Mall, Koerner S124, Vancouver, BC V6T 2B5 Canada

**Keywords:** Spinal cord injury, Editorial review, Research integrity, Inclusivity, Peer review

## Abstract

**Background:**

Diversity among editorial boards and in the peer review process maximizes the likelihood that the dissemination of reported results is both relevant and respectful to readers and end users. Past studies have examined diversity among editorial board members and reviewers for factors such as gender, geographic location, and race, but limited research has explored the representation of people with disabilities. Here, we sought to understand the landscape of inclusivity of people with lived experience of spinal cord injury specifically in journals publishing papers (2012–2022) on their quality of life.

**Methods:**

An open and closed 12-question adaptive survey was disseminated to 31 journal editors over a one-month period beginning December 2022.

**Results:**

We received 10 fully completed and 5 partially completed survey responses (response rate 48%). Notwithstanding the small sample, over 50% (8/15) of respondents indicated that their journal review practices involve people with lived experience of spinal cord injury, signaling positive even if incomplete inclusivity practices. The most notable reported barriers to achieving this goal related to identifying and recruiting people with lived experience to serve in the review and editorial process.

**Conclusions:**

In this study we found positive but incomplete trends toward inclusivity in journal practices involving people with lived experience of spinal cord injury. We recommend, therefore, that explicit and genuine efforts are directed toward recruitment through community-based channels. To improve representation even further, we suggest that editors and reviewers be offered the opportunity to self-identify as living with a disability without discrimination or bias.

## Background

Journal editorial boards, editors, and reviewers have been described as gatekeepers of knowledge [1], influencing the landscape of published research. By broadening the scope of publishable material, editors and reviewers with diverse backgrounds, perspectives, and life experiences construct an equitable peer review [1–3] and mitigate the influence of social bias, inaccessible infrastructure, and discrimination in the process [4, 5]. Biases arising from the exclusion of the perspectives and opinions of an affected community can lead to an undervaluing of the research, and the ultimate goal of improving patient outcomes [6].

Studies have investigated the composition of editorial boards for journals publishing articles in science, medicine, and public health disciplines in the context of representation of identity factors such as gender [7–9], race [10], and geographic location [2, 11]. These studies have highlighted the disparity in representation of women, people from marginalized communities, and non-US-based researchers in editorial positions, especially in leadership roles. A recent study led by the Journal of Medical Library Association [3] found that 80% of 46 editorial board members and 65% of 162 reviewers did not identify as having a disability or impairment. There have also been recent developments by publishers such as Lancet Psychiatry (Elsevier Press) that have subscribed to the goal of addressing barriers to participation and enhancing diversity across the publication landscape. In 2021, the editorial team announced a program devoted to providing opportunities for editors from resource-restricted regions and people living with mental health disorders [12].

Limited research, however, has specifically examined the representation of people with disabilities and spinal cord injury (SCI) specifically, which is the focus of the present work. Spinal cord injury affects between 250,000 and 500,000 people worldwide each year. The global unemployment rate of people with SCI can be as high as 60% [13] and between 18% and 35% in the USA alone [14]. Twenty-five to 45% of people with lived experience (PWLE) of SCI in the USA have a college degree [14]. Factors that can affect employment or professional service after SCI are the level of injury, limited mobility, and medical complications [15].

Researchers are increasingly engaging people with the lived experience of SCI in the design and translation of research that directly impacts them [16, 17]. With greater attention than before to PWLE’s priorities such as sexual health [18] and community reintegration [19], the goal is to maximize the benefits of resources and funding [20, 21]. At the same time, funding organizations such as the Congressionally Directed Medical Research Programs [22] and International Alliance of Mental Health Research Funders (IAMHRF) [23] are providing financial compensation for expenses related to participation in the research design process, paying honoraria and consultancies fees, requiring that researchers adopt co-created study designs that engage PWLE, and involving them in the grant review process.

As part of the ethics arm of a consortium called Mend the Gap that is working to develop a biomaterial scaffold to treat SCI, we sought to assess the inclusion of the valuable perspectives of PWLE of SCI in the editorial and peer review process in the reporting of the results, and identify existing gaps and opportunities for advancing inclusivity in the specific arena of research pertaining to quality of life in publication.

## Methods

An open and closed 12-question adaptive survey was disseminated to 31 journal editors, querying the inclusion of PWLE of SCI in the peer-review and editorial process of their journals. The data reported in this study [24] are in adherence with the Checklist for Reporting Results of Internet E-Surveys (CHERRIES) [25].

### Recruitment 

Thirty-one (31) journals were identified from a prior literature review [26] in which Google Scholar and PubMed were mined with key search terms pertaining to SCI (e.g., spinal cord injury, spinal cord injury repair, paraplegia, tetraplegia, quadriplegia) and ethics (ethic, autonomy, patient values, patient priorities, patient preferences, patient experiences, decision-making, quality of life, coping, adjustment, acceptance, resilience) for the 10-year time frame 2012–2022. The final set of publications meeting inclusion criteria (*N* = 70) pertained to the perspectives, priorities, and experiences of people living with SCI. Editors-in-chief and editorial staff of the journals publishing these papers were invited through email on December 7, 2022 to complete the anonymous, voluntary, web-based survey using the Qualtrics platform. AY obtained the email addresses of potential board and staff members from the corresponding journal, university or company website, or LinkedIn. Two reminder emails were sent at two-week intervals on December 14, 2022 and January 3, 2023, and the survey was closed on January 7, 2023. The emails to potential participants included the information of the primary investigator (JI), the purpose of the study, the approximate length of time the survey would take to complete, and indicated that all data would be anonymized and reported in aggregate. Professional role, sex, and disability were the three variables to which respondents could optionally report. Consent was obtained by including the following statement at the beginning of the survey: “By completing this survey, you consent to the integration of your answers into the analysis and aggregated reporting of our findings”. No incentives were offered for survey completion.

### Survey development

The survey [24] was developed collaboratively by AN, JI, and AY and piloted internally among the research team. It was also shared with a subset of members of the Mend the Gap Ethics and Knowledge Translation (E&KT) Council, including KT experts and a person living with SCI, for their feedback. A combination of 12 open and closed adaptive questions were distributed over 5 screens and covered the representation of PWLE of SCI in editorial and review processes, stages of engagement, avenues for recruitment, and challenges or barriers to inclusivity. Respondents were invited to add open-ended comments at the end of the survey. Respondents were able to edit their responses using the Back button.

### Data and statistical analysis

Overall response rate for the survey was calculated by dividing the number of partially and fully-completed responses by the number of participants contacted. Responses to each individual question were calculated using the denominator of responses for that question alone. For example, if only 11 participants responded to the question “Do individuals with lived experience of SCI participate in the review of manuscripts submitted to your journal?”, 11 was the denominator for determining the proportion of yes and no answers. Based on the small size of the survey questions and responses, descriptive statistics were applied to the data. AN and TB reviewed comments for synthesis and reporting.

## Results

We received 10 fully completed and 5 partially completed survey responses yielding an overall 48% response rate (15/31); all partially and fully completed surveys were analyzed [24].

### Characteristics of respondents

Ninety-percent (9/10) of respondents were Editors-in-Chief, one (1/10; 10%) was an Associate Editor. Forty-percent (4/10) self-identified as male and 50% (5/10) as female; one respondent chose not to answer. Thirty-percent (3/10) self-identified as having a disability.

### Representation of individuals with SCI in the editorial and review process

**Fig. 1:**
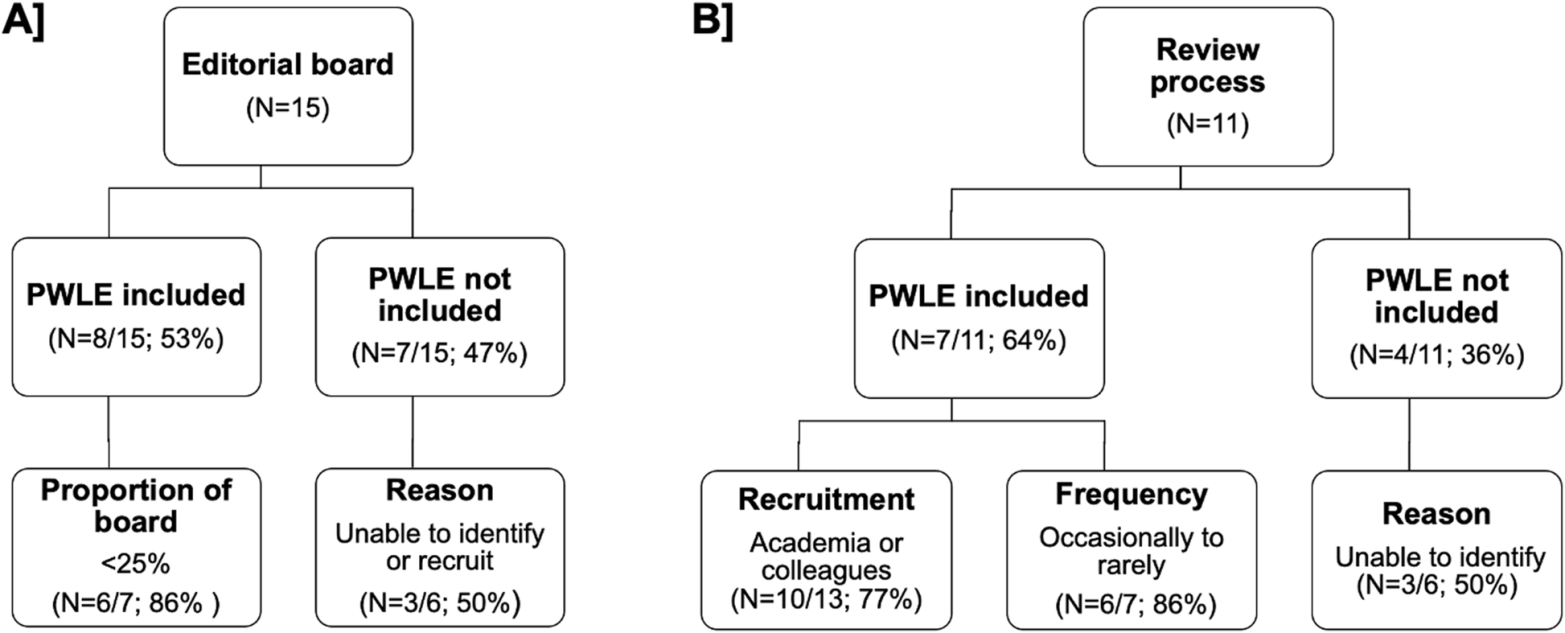
Summary of selected survey questions and findings regarding people with lived experience (PWLE) of spinal cord injury. Respondents reported on the inclusion of PWLE of SCI in the **A**] editorial process and **B**] review process. The most common method to recruitment to the review process is indicated in the Recruitment box. The frequency with which PWLE of SCI are engaged in the review process is indicated in the Frequency box. Explanations about lack of engagement is indicated in the Reason box

More than half of the respondents (8/15; 53%) reported the involvement of editors with lived experience of SCI. Overall, however, they represent less than 25% of the editorial boards. Seven of 11 (64%) respondents reported inclusion of PWLE of SCI or other disabilities in the peer-review process (Fig. 1).

### Stages of engagement

Most respondents (6/7; 86%) cited that they engage reviewers with SCI on occasional or rare bases. Two respondents elaborated that decisions about the assignment of reviewers to a specific review stage is based on research topic or level of expertise.

### Avenues for recruitment

Avenues for recruitment of reviewers with lived experience vary. They most frequently draw from academic channels and colleagues and collaborators (10/13; 77%). Three respondents also reported that advocacy or community groups, social media channels, and personal referrals are occasionally tapped for reviewer participation.

### Challenges or barriers to inclusivity

We received 6 open comments to the survey. They suggest that a main challenge to inclusion is the identification and recruitment of editors and reviewers living with a disability (Table 1).

**Table 1:** Comments about barriers and facilitators to inclusion of people with lived experience of spinal cord injury in editorial board and review processes

**Privacy and knowledge barriers to inclusion**	*"There may be reviewers for my journal who do not have SCI – I don’t ask for that information … I’m not sure I am even allowed to ask that."* *"We do not ask reviewers what their abilities or disabilities may be. I have no idea if we have anyone with a lived experience of SCI reviewing for us."* *"I do not even know whether there are reviewers with SCI."*
**Strategies for inclusion**	*"We are currently conducting research into how consumers, including people with SCI, would like to be involved with the journal. We did not think it appropriate for us to make the decisions in isolation."*
**Momentum toward inclusion**	*"Our journal is comprised of approximately 30–40% people who self-identify as disabled. They are not required to disclose what disability they have, so we don’t know what everyone has. Also, our journal focuses on a wide range of disabilities, so a wide range of conditions are represented on our editorial board and reviewers."* *"I believe that the inclusion of individuals living with SCI and other neurological disorders should be involved in all aspects of scientific reviews."*

## Discussion and conclusions

In this study, we aimed to identify existing gaps and opportunities for advancing inclusivity by surveying the involvement of PWLE of SCI in review and editorial practices in journals that publish research pertaining to quality of life (Table 2). The Canadian Institutes of Health Research (CIHR) recognizes that engaging patients, their families, and caregivers in research pertaining to them leads to better patient outcomes and has initiated the Strategy for Patient-Oriented Research (SPOR) to realize this goal [27]. In this small study, we found a positive, however incomplete, trend toward inclusivity. From the pool of respondents, half indicated that their journal review practices, the editorial process, or both involve PWLE of SCI. Respondents indicated that identifying and recruiting PWLE of SCI and other disabilities to participate in editorial and review processes is a challenge. Some survey respondents noted that they do not query the disability status of reviewers and, as a result, are not aware of the accurate representation of this population in the reviewer pool. Indeed, most academic journals do not request that reviewers disclose their disability status, and definitions of disability categories vary [28]. One respondent expressed that their journal is currently conducting research to determine the perspectives of end users, including PWLE of SCI, on preferred mechanisms of engagement.

**Table 2:** Gaps and opportunities for including people with lived experience (PWLE) of SCI in the review and editorial process

Gaps	Opportunities	Potential Concerns
Gaps in representation due to lack of knowledge about proper procedures around privacy.	Anonymized self-reporting of disability status; culture shift toward inclusivity over past discriminatory practices.	Deeply ingrained, historical practices are hard to change.
Challenges in identifying and recruiting PWLE of SCI to participate in review and editorial processes.	Open avenues of recruitment to include advocacy and community-based organizations. Provide compensation and resources to enable participation.	Privacy Engagement with the community may be viewed as tokenistic. Little precedent of level and type of reimbursement of reciprocity in the context of SCI.
Lack of professional credentials required to participate in review and editorial processes, limiting recruitment to academic channels.	Include reviewers from beyond the academic community to comment on relevance and usability.	Participant fatigue Limited precedent

The data also indicate that recruitment to journal processes occurs mainly through academic institutions and among colleagues and collaborators. Requirements of reviewers often include specialized knowledge, advanced educational degrees, and referrals from other reviewers [29, 30]. These credentials may limit opportunities for PWLE of SCI or other disabilities to participate in the editorial and review process, as institutional and attitudinal barriers to higher education, such as stigma [31] and inaccessible infrastructure [32, 33], persist.

Equity, diversity, and inclusion have taken a central focus in research and academia in recent years [34]. Equity-centred approaches not only seek to reconcile past injustices but aim to broaden the scope and impact of scholarship by accounting for diverse perspectives, experiences, and expertise. In the context of SCI, researchers are increasingly engaging research-end users, for example, clinicians, policy-makers and PWLE [35], to bridge the gap between research and practice. Representation of people with disabilities in journal practices and policies, however, remains limited [3].

Non-tokenistic, concerted efforts to engage PWLE are necessary to inform best practices in journal editorial processes and to ensure research is disseminated with the appropriate representation. Efforts to address ableism and accessibility challenges [36] and foster inclusive and safe environments will serve to promote the inclusion of people with disabilities in academia and facilitate a more accurate understanding of current representation [37, 38]. The standardised questionnaire developed by Elsevier [39] to determine diversity among reviewers pertaining to gender, ethnicity, and racial identity is a useful model as it provides opportunities for anonymous self-identification. Journals may also consider investing in greater recruitment efforts from advocacy and community-based organizations, and connecting with reviewers with lived experience of SCI who do not have academic backgrounds, much like research ethics boards, to comment on the relevance and usability of research for the community. Providing specific resources to support community members, acknowledging participants for their time and contributions, and permitting flexibility are all factors that should be considered to support meaningful engagement in the review process [40, 41].

We acknowledge the limitations of the small participant sample, and that respondents were drawn from journals publishing manuscripts pertaining only to quality of life in SCI and from only the most recent decade. Nonetheless, we deliver a first picture of the inclusion of PWLE of SCI in editorial and journal review processes of one sector of research that they have identified as most critical to them [26].

Journal editors and reviewers influence the landscape of published research. Here we found largely positive attitudes toward inclusivity and the involvement of PWLE of SCI in journal practices. Journals may address barriers to inclusion by creating opportunities for editors and reviewers to self-identify as having a disability, increasing recruitment and community engagement through relevant channels, and refreshing the make-up of their editorial boards as needed.

## Data Availability

The data supporting the findings of this study, including all relevant raw data, is available at [24].
